# Antitumour and antiangiogenic effects of Aplidin® in the 5TMM syngeneic models of multiple myeloma

**DOI:** 10.1038/sj.bjc.6604388

**Published:** 2008-06-03

**Authors:** J Caers, E Menu, H De Raeve, D Lepage, E Van Valckenborgh, B Van Camp, E Alvarez, K Vanderkerken

**Affiliations:** 1 Laboratory of Hematology and Immunology, Vrije Universiteit Brussel, Laarbeeklaan 103, Jette, Brussels 1090, Belgium; 2 Department of Pathology, UZBrussel 1090, Belgium; 3 PharmaMar USA, 64 Sidney Street, Cambridge, MA 02139, USA

**Keywords:** multiple myeloma, Aplidin, murine models

## Abstract

Aplidin® is an antitumour drug, currently undergoing phase II evaluation in different haematological and solid tumours. In this study, we analysed the antimyeloma effects of Aplidin in the syngeneic 5T33MM model, which is representable for the human disease. *In vitro*, Aplidin inhibited 5T33MMvv DNA synthesis with an IC_50_ of 3.87 nM. On cell-cycle progression, the drug induced an arrest in transition from G0/G1 to S phase, while Western blot showed a decreased cyclin D1 and CDK4 expression. Furthermore, Aplidin induced apoptosis by lowering the mitochondrial membrane potential, by inducing cytochrome *c* release and by activating caspase-9 and caspase-3. For the *in vivo* experiment, 5T33MM-injected C57Bl/KaLwRij mice were intraperitoneally treated with vehicle or Aplidin (90 *μ*g kg^−1^ daily). Chronic treatment with Aplidin was well tolerated and reduced serum paraprotein concentration by 42% (*P*<0.001), while BM invasion with myeloma cells was decreased by 35% (*P*<0.001). Aplidin also reduced the myeloma-associated angiogenesis to basal values. This antiangiogenic effect was confirmed *in vitro* and explained by inhibition of endothelial cell proliferation and vessel formation. These data indicate that Aplidin is well tolerated *in vivo* and its antitumour and antiangiogenic effects support the use of the drug in multiple myeloma.

Multiple myeloma (MM) is a plasma-cell malignancy characterised by accumulation of monoclonal plasma cells in the bone marrow (BM) and production of large amounts of monoclonal immunoglobulin or paraprotein. Multiple myeloma disease progression has been recognised as the result of different acquired changes in plasma-cell behaviour (self-sufficiency in growth signals, evasion of apoptosis and acquisition of invasive and spreading capacities) combined with an evolving crosstalk between myeloma cells and different cell types within the BM microenvironment (Hanahan and Weinberg, 2000; Mueller and Fusenig, 2004; Sirohi and Powles, 2004). Myeloma cells activate fibroblasts to secrete different growth factors, endothelial cells to initiate an angiogenic response, they stimulate immune and inflammatory cells and finally disrupt the balance between osteoblasts and osteoclasts, which results in osteolysis (Roodman, 2002; De Raeve and Vanderkerken, 2005).

The disease remains incurable for most patients, but advances in transplantation, the introduction of new compounds such as bortezomib (Richardson *et al*, 2005), thalidomide (Palumbo *et al*, 2006) and lenalidomide (Richardson *et al*, 2006), and the implementation of supportive agents (erythropoietin, bisphosphonates) have led to significant advances in delaying morbidity and mortality of the disease. Double autologous transplantation clearly provides an overall survival advantage, with an extended disease-free survival, while reduced-dose-intensity allogeneic transplantation has opened the door to many more patients for this treatment option (Kroger *et al*, 2002; Attal *et al*, 2003; Harousseau *et al*, 2005). The recent progress in unravelling the biology of the disease, particularly the intracellular pathways and the interaction with the microenvironment, has resulted in development of novel targeted therapies and therapeutic regimens with more disease specificity and less systemic toxicity (Anderson, 2007).

Aplidin® is such an antitumour drug, which was originally isolated from the marine tunicate Aplidium albicans and currently obtained by total synthesis. Aplidin was initially selected for its enhanced cytotoxicity against different tumour cell lines and its lower myelotoxicity. Aplidin is an extremely potent inducer of apoptosis, with concentrations that caused 50% inhibition of tumour growth (IC_50_) *in vitro* in a low nanomolar range, and it also exhibits strong antitumor activity in xenograft models (Urdiales *et al*, 1996; Erba *et al*, 2002; Broggini *et al*, 2003; Biscardi *et al*, 2005). In different haematological malignancies, such as acute lymphoblastic leukaemia, acute myeloid leukaemia and lymphoma, the drug is cytotoxic on primary human cells and cell lines, even on leukaemic cells carrying cytogenetic abnormalities that have poor prognostic implications (Bresters *et al*, 2003; Erba *et al*, 2003; Gajate *et al*, 2003). In addition to the cytotoxic properties of Aplidin, the molecule exhibits also strong antiangiogenic activity. The elucidation of direct effect against the VEGF loop has also been studied in several preclinical *in vitro* and *in vivo* models (Taraboletti *et al*, 2004). The results of these studies show that Aplidin reduces secretion of VEGF from MOLT-4 human leukaemia cells *in vitro* and has been shown to reduce the expression of VEGFR-1 in the same cell line (Broggini *et al*, 2003; Biscardi *et al*, 2005).

In the current study, we explored the *in vitro* and *in vivo* activity of Aplidin against MM cells. In the past years, our group examined the pathogenesis of MM by using the 5TMM murine myeloma models that were valuable in identification of biological processes and new therapeutic targets. Compared with xenograft models, using subcutaneously implanted cells from human myeloma cell lines that grow *in vitro* in a stroma-independent manner, these models are characterised by myeloma development in the bone marrow (Caers *et al*, 2005). This is a clinically important feature as the BM environment is strongly implicated in the development of drug-resistance (Damiano *et al*, 1999; Dalton, 2003). In addition to *in vivo* efforts, we also aimed to characterise some of the intracellular processes involved in the antitumor activity of Aplidin.

## MATERIALS AND METHODS

### Murine 5T33MM myeloma model

The 5T33MM cells originated spontaneously in ageing C57BL/KaLwRij mice and have since been propagated by intravenous transfer of the diseased marrow into young syngeneic mice (Radl *et al*, 1988). The model was continued by intravenous injection of five 10^5^ diseased BM cells into young (6- to 10-weeks old) syngeneic recipients (Harlan, Horst, the Netherlands) as described previously (Vanderkerken *et al*, 1997). The mice were housed and treated following the standards required by the UKCCCR Guidelines (UKCCCR, 1998). The 5T33MM*vivo* (5T33MM*vv*) cells grow *in vitro* in a stroma-dependent manner, with a survival of only a few days. The 5T33MM*vitro* (5T33MM*vt*) is a clonally identical variant that originated spontaneously from such an *in vitro* culture of 5T33MM*vv* cells (Manning *et al*, 1992). This myeloma cell line grows *in vitro* in a stroma-independent manner and is cultured and maintained in RPMI-1640 (Biowhittaker, Verviers, Belgium) supplemented with 10% bovine serum (Fetal clone I; Hyclone, Logan, UK), 1% natriumpyruvate, 100 U ml^−1^ penicillin, 100 *μ*g ml^−1^ streptomycin, 2 mM L-glutamine and 1% MEM (all from Biowhittaker). The BM endothelial cell line STR-10 was kindly provided by Dr M Kobayashi and also cultured in supplemented RPMI-1640 (Imai *et al*, 1999). To isolate the rat aortic rings, male Wistar rats were used and also treated according to Institution's Guidelines for the Care and Use of Laboratory Animals in Research. Approval of the Institutes′ Ethical Committee for Animal Care was obtained to perform these studies.

### Proliferation assay

*In vitro* proliferation was assessed by measuring DNA synthesis in a ^3^H-labelled thymidine incorporation assay. The cells were kept in serum-free medium RPMI-1640 and were treated with Aplidin at different concentrations for 17 h, while the vehicle solution was used as negative control. At 16 h before harvesting, cells were pulsed with 1 *μ*Ci (methyl ^3^H) thymidine (Amersham, Buckinghamshire, UK). Cells were harvested on paper filters (Filtermat A; Wallac, Turku, Finland) using a cell harvester (Inotech, Wohlen, Switzerland). The filters were dried for 1 h in an oven heated to 60°C and sealed in sample bags (Wallac) containing 4 ml of Optiscint Scintillation Liquid (Wallac). Radioactivity was counted using a 1450 Microbeta Liquid Scintillation Counter (Wallac). Results are expressed as percentages of the relative DNA synthesis: the amount of radioactivity (c.p.m.) of the vehicle treated cells was set to 100% and the decrease of the radioactivity of the treated cells compared with this is shown.

### Apoptosis assays

Apoptosis was assessed by FACS analysis of annexin V binding, caspase-3 cleavage, mitochondrial membrane integrity and cytochrome *c* release. To demonstrate the involvement of caspase activation in mediating the apoptotic effects of Aplidin, 5T33MMvt cells were preincubated for 30 min with the broad-spectrum caspase inhibitor, Boc-D-FMK (Merck Biosciences, Darmstadt, Germany) prior to treatment with Aplidin. After overnight incubation with Aplidin (20, 10 and 5 nM) cells were harvested and fixed for 10 min in 4.5% formaldehyde and 22% (v/v) acetone in FACSflow (BD Biosciences, Erembodegem, Belgium) and subsequently permeabilised with 1% saponin and 10% BSA in FACS flow and incubated with an FITC-conjugated rabbit anti-active caspase-3 antibody (BD Biosciences). Mitochondrial membrane integrity was analysed using the fluorescent dye DiIC1 (Invitrogen, Merelbeke, Belgium) following the manufacturers' instructions. Cytochrome *c* release from mitochondria was finally analysed following a protocol from (Waterhouse and Trapani, 2003). This assay is based on the principle that permeabilisation of cells will allow cytoplasmic cytochrome *c* to diffuse out of the cells. Cells were initially permeabilised and subsequently fixed and incubated with an FITC-conjugated monoclonal anti-cytochrome *c* antibody (eBioscience, San Diego, USA). All flow cytometric analyses were performed on a FACSCANTO (BD Biosciences, San Jose CA, USA) system using the FACSDIVA software.

### Western blot

After a 16-h incubation with different concentrations of Aplidin, cells were harvested and the cell pellets were lysed in lysis buffer containing 50 mM Tris, 150 mM NaCl, 1% NP-40 and 0.25% sodium deoxycholate. The following protease and phosphatase inhibitors were added: 4 mM Na_3_VO_4_ (Sigma-Aldrich, Bornem, Belgium), 1 mM Na_4_P_2_O_7_ (Sigma-Aldrich), 50 mM NaF (VWR, Leuven, Belgium), 5 mM EDTA (VWR), 1 mM AEBSF (ICN, Costa Mesa, CA, USA), 2 mg ml^−1^ aprotinin (Sigma-Aldrich), 50 mg ml^−1^ leupeptin (Sigma-Aldrich), 50 mg ml^−1^ pepstatin A (ICN), 500 mg ml^−1^ trypsin inhibitor (Sigma-Aldrich), 10 mM benzamidin (Sigma-Aldrich) and 2.5 mM pnp benzoate (Sigma-Aldrich). The cells were then cleared by centrifugation (5 min, 13 000 **g**) and sample buffer was added. After boiling, the samples were separated on a 10% SDS–PAGE gel and transferred to PVDF membranes (Bio Rad, Hercules, CA, USA). The membranes were blocked with PBS-containing 5% low-fat milk and 0.1% Tween 20 and probed with the appropriate antibodies, namely anti-caspase-8 and anti-caspase-9 (Cell Signaling Technology, Bioké, Leiden, the Netherlands), anti-cyclin D1 (eBioscience), cyclin D2 (Santa Cruz Biotechnology, Santa Cruz, CA, USA), cyclin-dependent kinase 2, 4 and 6 (all from Santa Cruz Biotechnology). For measuring total protein levels, the blots were stripped and reprobed with anti-*β*-actin (Santa Cruz Biotechnology). The horseradish peroxidase (HRP)-coupled secondary antibodies donkey anti-rabbit and goat anti-mouse antibodies were acquired from Amersham. Bands were visualised using the ECL system (Perkin Elmer, Zaventem, Belgium). Photographs and optical densities of the bands were analysed using KODAK software (Menu *et al*, 2004).

### Assessment of Angiogenesis by rat aortic ring assay

The rat aortic ring assay was performed as previously described (Van Valckenborgh *et al*, 2002). Briefly, thoracic aortas were removed from rats and sectioned into aortic rings of 1-mm long. The ring-shaped explants were then embedded in a rat-tail interstitial collagen (type 1) gel (Collagen R; Serva, Heidelberg, Germany) and allowed to polymerise in cylindrical agarose wells. Aortic rings (triplicates) were kept in culture at 37°C in 6 ml of medium conditioned for 48 h by 5T33MMvv cells in the presence of vehicle or 2.5 and 1.25 nM Aplidin. After 10 days, photomicrographs were captured using a Leica microscope and pictures taken with an Axiocam cold camera using AxioVision software. Images were recorded in triplicate. Image analysis was performed using the software Photoshop CS. After generation of a binary image, the number of microvessels, the maximal microvessel length, and the total number of branchings were determined manually (Van Valckenborgh *et al*, 2002).

### Aplidin treatment in the 5T33MMvv myeloma model

Three groups of 10 mice were given intravenous injections of 5T33MM cells; one group of 10 naive mice were included as negative control. Groups of 10 tumor-bearing mice received daily intraperitoneal treatment with either 90 or 60 *μ*g kg^−1^ Aplidin. A total of 90 *μ*g kg^−1^ was earlier found to be the maximum-tolerated dose by C57BL/KaLwRij mice. A similar group was treated with vehicle alone (cremophor/ethanol/water, dissolved in PBS). The treatment schedule consisted of 5 days of treatment, followed by 2 days of rest and was started in the 5T33MM model from injection of tumour cells onwards. Mice were weighed daily. When the vehicle controls showed signs of morbidity, all of them were killed. From one femur, BM cells were isolated to determine tumour load by FACS staining and cytosmear staining with May–Grünwald Giemsa; the other femur was fixed for immunohistochemical staining. Blood was harvested to determine serum paraprotein concentrations by capillary zone electrophoresis (Vanderkerken *et al*, 2005). To determine the effect of Aplidin, on survival, a similar experiment was performed. Two groups of 12 mice were given injections of 5T33MM cells: one group was treated with vehicle and the last group was daily treated with Aplidin, 90 *μ*g kg^−1^, intraperitoneally. Treatment continued until each animal showed signs of morbidity, namely, hind-limb paralysis, at which point they were killed. Tumour load was confirmed on BM samples.

### CD31 and Ki-67 staining on paraffin-embedded bone sections

The contralateral tibias and femora were incubated in zinc fixative (0.1 M Tris, 3 mM calcium acetate, 0.27 M zinc acetate, and 0.037 M zinc chloride), decalcified in FE10 (0.27 M EDTA, 0.3 M NaOH, 2% formalin), embedded in paraffin and 5 *μ*m sections were cut. Sections were stained for the presence of CD31 (PECAM-1) to identify the presence of microvessels or for Ki-67 to identify the proliferative cells (De Raeve *et al*, 2004). For CD31 retrieval, sections were incubated in trypsin to promote antigen retrieval and blocked with normal goat serum for 30 min. Sections were then incubated with a rat anti-CD31 antibody (PECAM-1; BD Biosciences), or an appropriate isotype control, at 4°C overnight. The sections were washed and incubated with a goat anti-rat antibody conjugated with biotin (1/100 dilution; BD Biosciences). The presence of bound antibody was detected with streptavidin–HRP conjugate in combination with tyramide signal amplification (NEN Life Science Products, Boston, MA, USA). Diaminobenzidine was used as substrate. The number of blood vessels and sinusoids (per 0.22 mm^2^) were counted in a tumour-infiltrated area with the highest microvessel density (hot spot). To measure proliferative activity of myeloma cells, immunohistochemical stainings with Ki-67 was used. Ki-67 antigen is expressed during the G1, S, G2 and M phases of the cell cycle, but is not expressed during the G0 (resting) phase. As Ki-67 antigen has a short half-life, it can be used as a marker of actively proliferating cells (Schluter *et al*, 1993). Sections from embedded tibia were incubated with a polyclonal anti-Ki-67 antibody (DakoCytomation A/S, Glostrup, Denmark) and subsequently visualised using the Envision+ detection systems (K4011, Rabbit DAB+; DakoCytomation). Ki-67-positive cells were counted at high magnification in an area with the highest proliferative activity. The percentage of Ki-67-positive cells was subsequently determined in this area at × 200 magnification by counting at least 400 nuclei.

### Statistical analysis

For statistical analysis of the *in vitro* data, Mann–Whitney test was used. For *in vivo* (antitumour) data, Student's *t*-test was used. For survival study, Kaplan–Meier analysis was performed. *P*-values smaller as 0.05 were considered significant. The IC_50_ concentrations were calculated using nonlinear regression analysis on the results obtained from the proliferations assay. The Statistical Package for the Social Sciences (SPSS) software v15.0 (SPSS Inc., Chicago, IL, USA) was used.

## RESULTS

**Figure 1:**
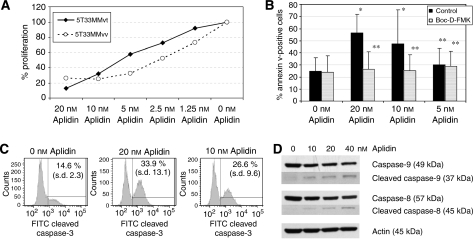
(**A**) Effect of Aplidin on 5T33MM DNA synthesis. (**B**) Effect of Aplidin on apoptosis induction. (**C**) Effect of Aplidin on activation of caspase-3. (**D**) Effect of Aplidin on caspace-8 and caspace-9 cleavage. Aplidin inhibits 5T33MM cell proliferation and induces apoptosis through caspase-3 and caspase-9 cleavage. Aplidin inhibits 5T33MM*vivo* and 5T33MM*vitro* cell proliferation at low nanomolar concentrations as measured by ^3^H-labelled thymidine uptake. The IC_50_ to inhibit 5T33MM*vivo* and 5T33MM*vitro* proliferation were 3.7 and 7.05 nM, respectively (**A**). On apoptosis, Aplidin significantly induced apoptosis at 10 and 20 nM. When 5T33MM*vt* cells were incubated with the broad-spectrum caspase inhibitor BOC-D-FMK, no apoptosis induction could be seen (**B**, ^*^P<0.05; ^**^P>0.05). Flow cytometry further revealed the activation of caspase-3 (**C**). Incubation with 10 nM Aplidin resulted in 26.6% activation of caspase-3, compared with 14.6% in control conditions. Increasing the concentration of Aplidin to 20 nM, resulted in an increase of caspase-3-positive cells 33.9%. Both differences were statistically significant (*P*<0.05), with results shown as the mean of three independent assays. Cleavage of other caspases was evaluated by Western blotting, that showed increased levels of cleavage products of both caspase-9 (39 kDa) and caspase-8 (45 kDa), and decreased levels of full-length caspase-9 (49 kDa) and caspase-8 (45 kDa). Equal protein loading was verified by *β*-actin staining.

We first investigated the effect of Aplidin on the growth of 5T33MM*vt* and 5T33MM*vv* cells *in vitro* by measuring ^3^H-labelled thymidine uptake. Treatment of these cells with Aplidin induced a dose-dependent decrease in cell proliferation compared with proliferation of the respective untreated cells. The mean Aplidin concentrations that caused 50% inhibition of growth (IC_50_) after 17 h of treatment were, respectively, 7.10 nM (95% confidence interval (CI)=6.87–9.17 nM) for 5T33MM*vt* cells and 3.87 nM (95% (CI)=0.021to 3.90 nM) for 5T33MM*vv* cells (Figure 1A).

**Figure 2:**
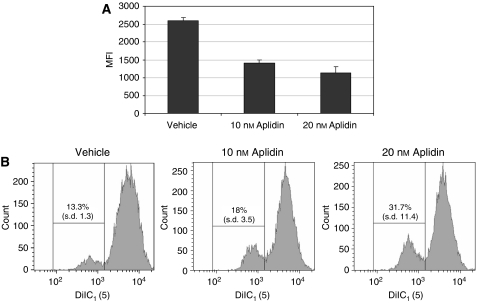
(**A**) Cytochrome *c* content and release by Aplidin treatment. (**B**) Mitochondrial membrane integrity and disruption by Aplidin treatment. Aplidin disrupts the mitochondrial membrane resulting in cytochrome *c* release. Mitochondrial membrane integrity and cytochrome *c* release were both analysed by flow cytometry. Panel **A** shows a graph summarising three independent assays. Incubation with 10 and 20 nM Aplidin reduced the fluorescence intensity of 5T33MM*vt* cells, indicating a decreased intracellular presence of cytochrome *c*. Mitochondrial membrane integrity was assessed using the dye DiIC_1_ that stains intact mitochondrial membranes, while disrupted membranes have decreased fluorescence. Incubation with 10 or 20 nM Aplidin increases the percentages of cells with lowered fluorescence (apoptotic) with 18 and 31.7% compared with 13.3% in control conditions.

To determine whether the reduction in proliferation of 5T33MM cells was accompanied by induction of apoptosis, we used annexin V-staining. 5T33MM*vt* cells were treated with different concentrations Aplidin (20, 10 and 5 nM) for 18 h and stained with annexin V and for activated caspase-3. A total of 56.7% (s.d.: 15.4) of the cell population treated with 20 nM Aplidin was annexin V-positive (i.e. apoptotic), compared with 24.7% (s.d. 11.34) of cells cultured in control culture conditions. An increase in apoptotic rate could also be observed at 10 and 5-nM concentrations (Figure 1B). Induction of apoptosis may involve activation of aspartate-specific cysteine proteases or caspases. 5T33MM*vt* cells treated with different concentrations of Aplidin were stained for activated caspase-3. As shown in Figure 1, caspase-3 was activated in a dose-dependent manner (Figure 1C). Preincubation with a broad-spectrum caspase inhibitor Boc-D-FMK resulted in inhibition of the apoptotic effects of Aplidin (Figure 1B), which proves the involvement of caspase activity in mediating the effects of Aplidin. Western blotting (Figure 1D) showed appearance of cleavage products of both caspase-8 and caspase-9 after incubation with Aplidin, indicating involvement of both the intrinsic and extrinsic apoptotic pathway in mediating the effects of Aplidin on apoptosis induction. As caspase-9 is activated through the intrinsic pathway, initiated by the disruption of the mitochondrial membrane and cytochrome *c* release, we evaluated mitochondrial membrane function and release of cytochrome *c* in Aplidin-treated 5T33MM*vt* myeloma cells. After incubation with the mitochondrial binding probe DiIC1, the percentage of cells with a lower fluorescence was higher in Aplidin-treated cells, indicating altered mitochondrial membrane potential (Figure 2B). After staining with an antibody recognising cytochrome *c*, the mean fluorescence (MF) of Aplidin-treated cells was lower compared with the MF of non-treated cells (Figure 2A).

**Figure 3:**
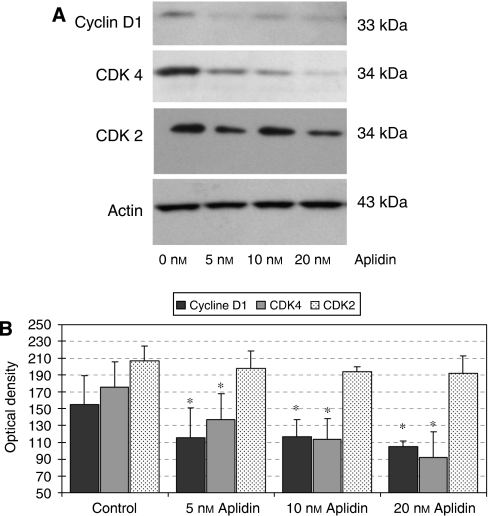
(**A**) Western blots of cell-cycle regulators affected by Aplidin. (**B**) Optical densities of bands seen in Panel **A**. Aplidin causes an arrest in G0/G1 phase by decreasing the expression of cyclin D1 and CDK4. Western blotting of the different proteins involved at cell-cycle progression at G0 to G1 and G1 to S phase revealed decreased expression of cyclin D1 and CDK4. These effects were concentration dependent, as higher concentrations further decreased the expression. ^*^P<0.05.

**Figure 4:**
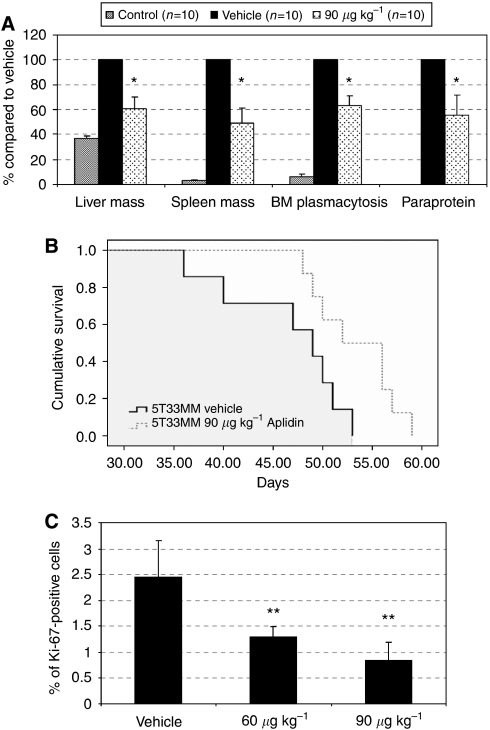
(**A**) Effect of Aplidin on different myeloma parameters of treated mice. (**B**) Effect of Aplidin on the survival of 5T33MM diseased mice. (**C**) Quantification of Ki-67-positive 5T33MMvv cells in the treatment groups. Aplidin treatment reduced tumor load in the 5T33MMvv diseased mice and decreased proliferation *in vivo*. 5T33MMvv diseased mice were treated according to the schedules described under Materials and Methods. On the different tumor parameters, we noted significant effects on paraprotein concentrations in peripheral blood, bone marrow plasmacytosis and spleen and liver mass (**A**, ^*^P<0.001). The results shown are the mean of 10 mice in each treatment group. The experiment was performed in duplicate with similar results on endpoints. Aplidin treatment also prolonged the survival of 5T33MMvv mice (**B**); the mean survival of vehicle-treated mice was 42.9 days, compared with 51.5 days for Aplidin-treated mice (*P*<0.05). *In vivo*, cell-cycle progression and myeloma cell proliferation were analyzed by Ki-67 staining. Aplidin treatment decreased the nuclear expression of Ki-67, suggesting a lowered proliferative index and less cells in cell-cycle progression (**C**, ^**^P<0.05).

On cell-cycle progression, Aplidin exposure resulted in an increase in the percentage of 5T33MM*vt* cells in G0/G1 phase (65.6 *vs* 57.5%) and a decrease in the S and M phases (33.3 *vs* 23.1%, results not shown). Knowing that the transition from G1 to S phase is regulated by the cyclin D1, cyclin D2 and cyclin-dependent kinase 2 (CDK2), CDK4 and CDK6 complex, we performed Western blot of these proteins. Aplidin exposure resulted in a decrease in protein expression of both cyclin D1 and CDK4, while concentrations of actin (as control protein) remained unaffected (Figure 3). CDK2 was affected only slightly. These data indicate that Aplidin inhibits 5T33MM cell proliferation at low nanomolar concentrations by causing arrest in G0/G1 by affecting the expression of cyclin D1 and CDK4. Cell-cycle progression was also monitored *in vivo* by using immunohistochemical staining for Ki-67. As in the human disease (Witzig *et al*, 1999), murine 5T33MM*vv* have a low proliferative index, which yielded in a low percentage of Ki-67-positive myeloma cells. Intraperitoneal treatment with Aplidin decreased the number of Ki-67-positive cells (Figure 4B), indicating an *in vivo* confirmation of the *in vitro* effects of proliferation and cell-cycle progression.

Following *in vitro* studies, *in vivo* intraperitoneal treatment with maximum tolerated doses of Aplidin resulted in decreased tumour load. In Figure 4A, the effect of Aplidin on paraprotein and tumour load in the BM is shown. Mice treated with Aplidin at 90 *μ*g kg^−1^ showed a 42% reduction in serum paraprotein concentrations and a 35% reduction in the percentage of 5T33MM idiotype-positive cells in the BM (*P*<0.001). In this model, MM cells also accumulate in both the spleen and liver. Treatment with Aplidin reduced overall splenomegaly by 60% and hepatomegaly by 40% (*P*-values <0.001). As Aplidin treatment inhibited tumour progression and angiogenesis, we next studied its effect on the overall survival of the mice by Kaplan–Meier analysis (Figure 4B). The mice were treated either with the vehicle or with Aplidin. The Aplidin treated mice had a prolonged survival (51.5 days as compared with 42.9 days treated with vehicle only, *P*<0.05) according to Kaplan–Meier analysis. In the past years, other agents have been administrated to 5TMM-bearing mice to study their potential effect on survival. Most agents (zoledronic acid, the CDK4/6 inhibitor PD 0332991, the IGF-1R tyrosine kinase inhibitor picropodophyllin and recombinant osteoprotegerin) showed similar effects (1.2 to 1.5 × prolongation) in survival studies, as the results obtained with Aplidin (Croucher *et al*, 2003; Vanderkerken *et al*, 2003; Menu *et al*, 2006, 2007). Only the survival rates obtained with the p38 MAP Kinase inhibitor SCIO-469 (which were three times increased) were superior to these results (Vanderkerken *et al*, 2007).

**Figure 5:**
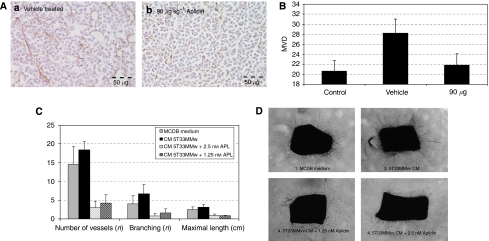
(**A**) CD31 immunostaining on bone marrow sections. (**B**) Quantification of microvessel density (MVD). (**C**) Quantification of rat aortic ring assays. (**D**) Images from a rat aortic ring assay. The myeloma-associated angiogenesis could be inhibited by Aplidin *in vitro* and *in vivo*. Panel **A** illustrates the CD31 immunostainings on the BM sections of 5T33MM diseased mice, which were treated with the vehicle solution or with Aplidin, 90 *μ*g kg^−1^. Quantification of the MVD on the BM sections of the different treatment groups revealed a decreased MVD in both treatment groups compared with the group treated with the vehicle solution (**B**). *In vitro* Aplidin inhibited new vessel formation in a rat aortic ring assay. Incubation with conditioned medium obtained from 5T33MM*vv* cells increased the total number, branches and maximal length of the newly formed vessels. Incubation with 2.5 and 1.25 nM Aplidin inhibited the vessel formation. Four representative pictures are shown in panel **D** and quantifications are shown in panel **C**. Each assay was run in triplicate and the results are the mean±s.d. of three independent assays.

Next to analysing the antimyeloma effects of Aplidin, we also analysed the antiangiogenic effects of Aplidin. We have previously shown that 5T33MM cells stimulate angiogenesis *in vivo*, as assessed by quantifying the MVD (Van Valckenborgh *et al*, 2002). Immunohistochemical staining for CD31 (Figure 5A and B) demonstrated the increased MVD in tumour-bearing mice. When mice were treated with Aplidin, the MVD was reduced back to control levels. Angiogenesis is a multistep process, in which quiescent endothelial cells are stimulated by angiogenic factors to proliferate, migrate, invade the underlying matrix, form capillary-like tubular structures and, finally, organise a network of mature, functional blood vessels (Carmeliet, 2005). To find correct concentrations to target angiogenesis *in vitro,* the BM endothelial cell line STR-10 was treated with different concentrations of Aplidin. The required concentrations to block endothelial cell proliferation (measured by ^3^H-labelled thymidine uptake) were lower with an IC_50_ of 2.68 nM (95% (CI)=2.23–2.74 nM) than the concentrations required to affect myeloma cell proliferation (results not shown). The rat aortic ring assay consists of an organ culture assay in which microvessel development is a net result from endothelial cell proliferation, migration and capillary formation. Conditioned medium of 5T33MM*vv* cells is known to increase this process, resulting in a network of small vessels with multiple branches (Figure 5D; Van Valckenborgh *et al*, 2002). When Aplidin (2.5–1.25 nM) was added to this conditioned medium, vessel outgrowth could not be detected. These differences were statistically significant (*P*<0.05). Aplidin added to aortic rings cultured under basal conditions also inhibited vessel formation, indicating the strong antiangiogenic capacities of the compound. At lower concentrations (0.6 and 0.3 pM) Aplidin also remained active in inhibiting vessel formation, which could only be seen to reappear at 0.15 nM Aplidin (results not illustrated). This latter is 20 times lower as the IC_50_ of Aplidin on proliferation of 5T33MMvv cells.

## DISCUSSION

In the treatment of MM disease, an increasing number of therapeutic agents are becoming available, with the potential for significant symptom palliation, induction of disease responses and prolongation of disease-free survival. These different therapeutic approaches, including peripheral-blood stem cell transplantation and newer agents (thalidomide, bortezomib, and lenalidomide), have pronounced antimyeloma effects; but, unfortunately, MM disease often relapses. These approaches have only a modest impact on global patient overall survival in several randomised trials, and, as a consequence, there is still much need for improved antimyeloma therapies.

In the discovery of these therapies, preclinical mouse models may be exploited to accelerate drug development. They not only provide the opportunity for rapid validation of potential drug targets, but also help to derive critical variables such as achievable concentration of drug dose, routes of administration, frequency of dosing and toxicity profile. In our laboratory the syngeneic 5TMM models have been used in several studies to identify different biological processes in MM disease and validate the antimyeloma effects of different compounds (Menu *et al*, 2006; Vanderkerken *et al*, 2007). As Aplidin has pronounced anticancer activities *in vitro*, we were interested in testing the compound in this model, with the hope of identifying some of the intracellular pathways involved and help define optimal dosing schedules in our model.

The present report indicates that Aplidin has both *in vitro* and *in vivo* antimyeloma effects in the murine 5T33MM model. Studies involving the mechanism of action of Aplidin indicate that this compound acts through a complex number of events that affect proliferation, cell-cycle progression and apoptosis (Erba *et al*, 2002; Garcia-Fernandez *et al*, 2002). In 5T33MM*vt* cells, Aplidin induces a G0/G1 cell-cycle arrest. Analyses of the proteins implicated in the control of cell-cycle progression showed decreases in the expression of both cyclin D1 and CDK4. Translocation of the *cyclinD1* gene to the immunoglobulin heavy-chain locus t(11;14) and overexpression of cyclin D1 RNA have been implicated in cell-cycle dysregulation in MM (Bergsagel and Kuehl, 2001). The group of Chen-Kiang, however, showed that in the transition from G1 to S phase, retinoblastoma becomes phosphorylated by the exclusive pairing of CDK4 with cyclin D1 or CDK6 with cyclin D2 (Ely *et al*, 2005). The decreased expression of both CDK4 and cyclin D1 by Aplidin thus explains the arrest seen in cell-cycle progression.

Apart from affecting proliferation and cell-cycle progression, Aplidin also induces apoptosis in 5T33MM cells. This was characterised by annexin V staining cleavage of caspase-3, -8 and -9 and decreased mitochondrial membrane potential. The latter is often accompanied by increased permeability of the outer mitochondrial membrane, with release of intermembranous material to the cytosol, including several regulators of apoptosis. Flow cytometry demonstrated a significant release of cytochrome *c* from the mitochondria to the cytosol of 5T33MM cells exposed to Aplidin. Released cytochrome *c* is known to participate, together with procaspase-9 and apoptotic protease-activating factor, in the assembly of active apoptosomes, that in turn trigger the proteolytic cleavage of procaspase-3, with its ensuing activation (Riedl and Salvesen, 2007). In fact, evidence showing activation of caspase-3 and caspase-9 were present, indicating activation of the endogenous caspase-dependent cell death route. To confirm whether caspase activity really was required for cell death induced by Aplidin, the pancaspase inhibitor BOC-D-FMK was used to block apoptosis. These data shown indicate that Aplidin indeed induced BOC-D-FMK-sensitive cell death.

Previous works have demonstrated that activation of intrinsic apoptotic pathways through mitochondria is one of the hallmark activities of Aplidin (Garcia-Fernandez *et al*, 2002; Gajate *et al*, 2003). In the subsequent studies, the mitochondrial changes were shown to be caused by different interactions. The sustained activation and phosporylation of c-Jun N-terminal kinase (JNK) by reactive oxygen species (ROS) is recognised as one of the crucial mechanisms in the effects of Aplidin on breast cancer cells (Garcia-Fernandez *et al*, 2002; Cuadrado *et al*, 2004; Gonzalez-Santiago *et al*, 2006; Suarez *et al*, 2006). This ROS induction was found to be caused by an alteration in the glutathione homeostasis and by a rapid activation of the small GTP-binding protein Rac (Cuadrado *et al*, 2003; Gonzalez-Santiago *et al*, 2006). Next to phosphorylation of JNK, Aplidin also causes cleavage of Bid, a proapoptotic Bcl-2-family member that connects the Fas/CD95 cell death receptor (DR) to the mitochondrial apoptotic pathway, in human leukaemic cell lines (Gajate *et al*, 2003). Phosphorylation of JNK is currently being studied as *in vivo* pharmacodynamic marker of Aplidins' mechanisms of action. In this study, maximal activation of JNK was seen in tumour derived from Aplidin-treated animals 4–12 h post treatment, while phospho-JNK levels were lower at earlier and later times (24–48 h) (Muñoz *et al*, 2007).

In addition to the mitochondrial pathway, the DR pathway regulates apoptosis in myeloma cells. Binding of its ligands, such as Fas ligand, TNF-*α*, and TNF-related apoptosis-inducing factor, to DR on the plasma membrane activates its adaptor proteins and finally triggers proteolytic activation of caspase-8 (Oancea *et al*, 2004). It was earlier shown that adding an anti-Fas-blocking antibody partially inhibited the Aplidin-induced apoptotic response, suggesting that the apoptotic response induced by Aplidin in leukaemic cells is partially mediated through Fas/CD95 binding (Gajate *et al*, 2003). Aplidin acts by clustering of CD95, but also by additional DRs and membrane-bound FasL, together with downstream signalling molecules, into aggregated lipid rafts (Gajate and Mollinedo, 2005). Ocio *et al* (2007) recently showed that, in addition to JNK phosphorylation, Aplidin induces a translocation of Fas and CD95 translocation to these lipid rafts in human myeloma cells. Our results show both cleavage of caspase-8 and caspase-9, and thus indicate an involvement of both the intrinsic and extrinsic apoptotic pathways in mediating the effects of Aplidin.

Daily administration of Aplidin in mice did not influence body weight suggesting that the agent is well tolerated when administered on a daily basis. The schedule consisted of daily administration of Aplidin and resulted in a 42 and 35% reduction in paraprotein levels and BM plasmacytosis, respectively, in the 5T33MM model. These results are in the same range as the results obtained with a single treatment with melphalan (4 mg kg^−1^) in the same model. This treatment decreased paraprotein levels with the 44% and BM plasmacytosis with the 47% schedule. Also, when treatment was started after the first signs of disease manifestation in the 5T2MM model (*in casu* appearance of an M-spike in serum electrophoresis), Aplidin treatment significantly (*P*=0.030) decreased tumour burden, as measured by serum paraprotein concentrations in the 5T2MM model. Mean paraprotein concentration of diseased mice was 0.62 g dl^−1^ (s.d.: 0.44) and 0.19 g dl^−1^ (s.d.: 0.11) in vehicle- and Aplidin-treated mice, respectively (results not illustrated).

Of particular importance in MM disease is the lack of toxicity on haematopoesis. Alkylating chemotherapies are part of induction treatments in myeloma, but affect the haematopoietic stem cells, which might interfere with an autologous BM transplantation, which is crucial to obtain longer remission times in MM patients. Albella and co-workers showed that Aplidin inhibits colony formation by human BM cells at high concentrations (150–530 nM) *in vitro*. These concentrations are 20-fold higher than the maximal drug concentrations found in the plasma of patients, given the maximal tolerable dose and 15-fold higher than the concentrations required to inhibit cancer growth. The clinical toxicological data obtained in phase-I and phase-II studies do not show BM toxicity in the different treatment schedules used (Gomez *et al*, 2003).

Aplidin has previously been described as an antiangiogenic compound (Broggini *et al*, 2003; Biscardi *et al*, 2005). *In vitro* and *in vivo*, Aplidin inhibited the neovascularisation associated with MM disease. These results support the earlier hypothesis proposed by others that Aplidin exerts a potent antiangiogenic activity by having a direct effect on endothelial cells, in addition to inhibiting production of angiogenic factors (Taraboletti *et al*, 2004). As seen in this study, Aplidin inhibited, in our experiments, both the physiological as the 5T33MM*vv*-conditioned media-induced angiogenesis seen in a rat aortic ring assay.

In conclusion, administration of Aplidin resulted in strong antimyeloma activity in this syngeneic *in vivo* model. Aplidin clearly demonstrated activity against the various endpoints evaluated in this model (e.g. paraprotein, BM invasion, angiogenesis and so on). Besides the previously reported antimyeloma and antiangiogenesis effect, the present study indicates an additional direct effect on cell-cycle progression. Further studies are needed to define the relative effect of Aplidin on, respectively, the tumour compartment and endothelial cell compartment, and to identify optimal schedule and doses, to better exploit its multifaceted antineoplastic potential.
